# Assessing the compliance of accurately documenting medication history in CAMHS – completion of the audit cycle

**DOI:** 10.1192/bjo.2021.126

**Published:** 2021-06-18

**Authors:** Laura Guest, Irangani Mudiyanselage, Swetangi Ambekar, Sudheer Lankappa

**Affiliations:** 1 Nottinghamshire Healthcare NHS Foundation Trust; 2 Derbyshire Healthcare NHS FT

## Abstract

**Aims:**

To assess the documentation of medication across all Child and Adolescent Mental Health Service (CAMHS) teams in the south region of Derbyshire Healthcare NHS Foundation Trust against a locally agreed protocol. The aim is to ensure accurate and timely documentation of medication history in a standardised way to reduce the risk of medication errors.

**Method:**

We randomly selected 78 patients across seven teams within CAMHS that were currently prescribed medication as of November 2020. We reviewed each patient to see if medication history had been recorded in the specified section of the trust's patient database PARIS. We then cross referenced this information with the patient notes, clinic letters and prescriptions to review accuracy of information in terms of recording of drug name, dose, frequency, and whether the medication was regular or as required. We compared the data to the results of a previous audit in 2017 which used the same methods.

**Result:**

Of the 78 patients, 74% (n = 58) had medication recorded in the correct section of PARIS compared to 13% in the 2017 audit. We found that compliance varied between different CAMHS teams ranging from 0% to 100%. Of those with medication history recorded, 86% had all drug names listed correctly, 79% had all drugs listed at the correct dose, 71% had the correct frequency recorded and 81% had whether the medication was regular, or PRN recorded.

**Conclusion:**

Although we have seen improvement in standardised documentation of medication history since 2017, it remains difficult to rely on this information being up to date and reliable. There was a wide range of compliance in documentation of medication history across different teams, possibly reflecting how effectively the teaching following the previous 2017 audit had been delivered to each team. We have completed more teaching for medical and non-medical prescribers across all localities to highlight the importance of timely and standardised documentation. This is particularly important in CAMHS where the prescribing of medication often remains the responsibility of secondary care, with clinicians regularly prescribing on behalf of colleagues from other teams. Our findings support the move within the Trust towards a system where medication can be both documented and electronically prescribed in the same place (System One).

